# Effect of social condition on behavioral development during early adult phase in *Drosophila prolongata*

**DOI:** 10.1007/s10164-017-0524-x

**Published:** 2017-09-04

**Authors:** Takashi Matsuo

**Affiliations:** Department of Agricultural and Environmental Biology, The University of Tokyo, Yayoi 1-1-1, Bunkyo-ku, Tokyo, 113-8657 Japan

**Keywords:** Behavioral ontogeny, Courtship behavior, Social experience, Mating tactics, Anticipated male–male competition

## Abstract

Behavioral changes during early development provide useful insights into the internal mechanisms that generate complex behavior expressed by mature individuals. At the same time, social conditions during early adult phase can influence behavior in later stages of development even in holometabolous insects. In this study, age-dependent changes in courtship behavior and the effect of social conditions were examined in a fruit fly, *Drosophila prolongata*. Younger males showed lower mating activity and simpler courtship behavior. Mating activity reached a maximum level by 5 days after eclosion, whereas expression of complex courtship behavior was not yet fully developed at that time, suggesting that they are controlled by different mechanisms. When two males were maintained in the same vial, not only mating activity but also courtship complexity was reduced, demonstrating for the first time that preceding social experience, not current social conditions, influenced the complexity of male courtship. The effect of social experience was completely erased by 1 day of isolation, however, showing that social experience did not suppress or promote behavioral development itself. Rather, these results suggest that the observed effect of social experience was a plastic response of males that reduced investment in courtship effort by anticipating increased male–male competition.

## Introduction

Age-dependent changes in behavior (behavioral ontogeny) provide insights into the mechanisms underlying development of complex behavior. Even during the adult phase of holometabolous insects, changes are observed in various behaviors, such as aggression in the flesh fly (Moore et al. 2014), age-dependent division of labor in social insects (age polyethism; reviewed in Brian 2010), and mating behavior in *Drosophila* fruit flies (Boake and Adkins 1994; Pitnick et al. 1995; Moulin et al. 2001; Ruhmann et al. 2016; Ford et al. 1988; Long et al. 1980). In *Drosophila melanogaster*, social conditions during early adult phase are known to influence mating behavior in later stages; For example, males maintained at low density during the first 4 days of adult life acquired more mates than those maintained at high density in mate choice tests (Dukas and Mooers 2003). In another study, it was shown that previously mated males courted females better and outcompeted sexually inexperienced males for copulations (Saleem et al. 2014). The best studied case is the increase in copulation duration induced by exposure to rival males prior to mating (Bretman et al. 2009). However, much less attention has been paid to developmental changes in the complexity of courtship behavior and how social experience influences this. The reason could be partially because of the limited qualitative variation among individuals in the courtship behavior of most *Drosophila* species. Age-dependent changes have been observed only in quantitative aspects such as sound components made by wing vibration, vigor measured by courtship duration or courtship index, and copulation rate (Boake and Adkins 1994; Pitnick et al. 1995; Moulin et al. 2001; Ruhmann et al. 2016; Ford et al. 1988; Long et al. 1980).

*Drosophila prolongata*, a member of the *rhopaloa* subgroup of the *melanogaster* species group (estimated divergence time from *D. melanogaster* is 35 million years ago), is endemic to southwestern China, northeastern India, Myanmar, and Vietnam (Singh and Gupta 1977; Toda 1991; Setoguchi et al. 2014). The forelegs of *D. prolongata* are extraordinarily thick and elongated in males. Males of *D. prolongata* exhibit two types of courtship behavior. In the simpler form, males proceed from “wing vibration” to “rubbing” (stimulation of the female’s abdomen from behind using their forelegs) before attempting copulation. In the complex form, “leg vibration” occurs before rubbing, in which males stimulate the female’s body using their forelegs from in front of her (Setoguchi et al. 2015). Leg vibration requires quick repositioning of the male’s body relative to the female’s (wing vibration: side of the female → leg vibration: front → rubbing: behind) and coordinated movement of the forelegs. This is not observed in other *Drosophila* fruit flies, including the most closely related species, suggesting that leg vibration specifically evolved in the *D. prolongata* lineage (Setoguchi et al. 2014). Leg vibration increases female receptivity and is therefore observed more frequently when female receptivity is initially low (Setoguchi et al. 2015). Male genetic background can also influence usage of leg vibration, and some strains perform it more often than other strains irrespective of female receptivity (Kudo et al. 2015; Setoguchi et al. 2015). Variation in courtship behavior in *D. prolongata*, which is unambiguously and instantly recognized, provides a unique opportunity to examine age-dependent shifts from simple to complex courtship behavior in this species.

In this study, using *D. prolongata* courtship behavior, we examined: (1) age-dependent changes in leg vibration usage, and (2) effect of social experience on behavioral development during early adult phase. The following hypotheses were tested: (1) The complex form of courtship behavior requires a longer developmental period to be fully expressed than the simpler form; (2) Social experience influences the complexity of courtship behavior; (3) Social experience affects the developmental process of behavioral complexity, therefore its effect will not be erased within a short period.

## Materials and methods

### Fly strains

As male courtship behavior is influenced by external stimuli such as female responses, the experimental design was critical to disentangle the influence of external factors from the intrinsically generated form of male courtship behavior. In *D. prolongata*, males tend to perform more leg vibration against females with low receptivity, which require leg vibration to accept copulation (Setoguchi et al. 2015). To reduce the influence of female factors on male courtship behavior, SaPa010 was selected as the female tester strain (Kudo et al. 2015). Females of SaPa010 are highly receptive and accept copulation irrespective of how they are courted; i.e., leg vibration was not required, and the whole courtship duration could be as short as a few seconds (Setoguchi et al. 2015; Hitoshi et al. 2016a). With this characteristic, SaPa010 females are an amenable tester to detect differences in male courtship behavior, because they accept copulation even when young males perform immature forms of courtship. For males, another strain, BaVi043, was used because their courtship behavior is least influenced by females; even against highly receptive SaPa010 females, more than 70 % of mature BaVi043 males perform leg vibration (Setoguchi et al. 2015). Using the combination of SaPa010 females and BaVi043 males, intrinsically generated courtship structure was expected to be observed with the least interference from female receptivity. These strains were directly established from single females collected at Sa Pa (22°20′N, 103°52′E; September 2004) and Ba Vi (21°04′N, 105°22′E; March 2005) in Vietnam, and had been maintained as isofemale lines since then by more than 100 generations of inbreeding under laboratory condition with average population size of around 50 individuals (Kudo et al. 2015).

Flies were reared on standard cornmeal medium for *Drosophila* culture (Setoguchi et al. 2014). As development of *D. prolongata* is inhibited by higher temperature (Hitoshi et al. 2016b), all experiments and maintenance of cultures were carried out at 20 °C. A 12:12 h light:dark cycle was applied throughout the experiments.

### Observation of mating behavior

Preparation of the behavioral assay was the same as described previously (Setoguchi et al. 2015; Kudo et al. 2015; Hitoshi et al. 2016a; Kudo et al. 2017). Prior to observation, flies were starved for 1 day in vials containing a wet cotton ball to increase locomotor activity during observation. Courtship behavior was observed in a glass chamber (50 mm diameter × 70 mm height), the inner wall of which was treated with silicone polish to prevent the flies from climbing. A disc of wet filter paper was placed on the bottom of the chamber, and at the center of it was placed a “mating stage,” consisting of the lid of a 15-mL conical tube (23 mm diameter × 11 mm height) filled with *Drosophila* instant medium (Formula 4–24 *Drosophila* Medium, Carolina Biological Supply Co., Burlington, USA). Up to eight chambers were arranged in two rows, isolated from each other by paper partitions, and covered with glass plates. Approximately 5 min after introduction of a female, a male was introduced into the chamber. Mating behavior was recorded for 1 h using a digital video camera (HDR-CX720V; Sony, Tokyo, Japan) installed 80 cm above the chambers. All behavioral observations were conducted during the last 2 h of the light phase, because the highest locomotor activity was observed during either the first 2 h or last 2 h of the light phase in both sexes.

### Experimental conditions

#### Condition 1: isolated

To examine developmental changes in courtship behavior, newly emerged male flies were maintained individually in glass vials (25 mm diameter × 100 mm height, containing cornmeal medium). Observation of mating behavior was carried out 3, 5, or 7 days after eclosion. Each individual was used only once for observation. Females were always staged for 7 days after eclosion in a group of 10 individuals per vial.

#### Condition 2: social

To examine the effect of social experience on development of courtship behavior, two males were maintained in a vial for 3, 5, or 7 days after eclosion. Both males were used for behavioral observation separately (individually). Females were prepared in the same manner as in condition 1.

#### Condition 3: social → isolated

To examine whether the social effect is reversible, two males were first maintained in a vial for 2, 4, or 6 days after eclosion, then separated and maintained individually in vials for 1 day before behavioral observation. Females were prepared in the same manner as in condition 1.

### Data analysis

Videos were played back on a computer and inspected visually. Courtship rate was calculated as the proportion of males that exhibited any courtship (either one or a combination of tapping, leg display, wing vibration, leg vibration, and rubbing) towards the female. Copulation rate was calculated as the proportion of pairs that copulated within the observation period (1 h). The following variables were scored for copulated pairs: courtship duration, occurrence of leg vibration, and copulation duration. Courtship duration was the total time spent on any courtship behaviors: tapping, leg display, wing vibration, leg vibration, and rubbing. Leg vibration rate was calculated as the proportion of males that performed leg vibration prior to copulation among the copulated pairs. The count data (courtship rate, copulation rate, and leg vibration rate) were analyzed using generalized linear models (GLMs) with binomial distribution and logit link function, while the other data (courtship duration and copulation duration) were analyzed using GLMs with gamma distribution and log link function. Each model included “male age” and “experimental condition” as explanatory variables (in addition, the model for courtship duration included “occurrence of leg vibration”). The effect of each explanatory variable was assessed using a likelihood ratio test in which reduction in deviance by adding the focal explanatory variable to the corresponding reduced model was examined. All statistical analyses were performed using R v3.1.1 (R Core Team 2014).

## Results

### Development of courtship behavior

The effect of age was significant for all variables (Table 1). Results obtained from condition 1 illustrated behavioral development during early adult phase (Figs. 2, 3, 4, 5, 6, condition 1). Courtship rate was low in 3-day-old males (Fig. 2, condition 1); almost half of the males did not show any courtship even when they visually recognized the female, suggesting that their mating activity was not yet mature. On the other hand, nearly 100 % of 5- and 7-day-old males courted the females, suggesting that mating activity reached a maximum level by the age of 5 days. Note that the copulation rate was almost equal to the courtship rate (Figs. 2, 3), proving that SaPa010 females accepted most of the courting males, as expected.

**Table 1:** Results of statistical analysis using generalized linear models (GLMs)

Response variable	Explanatory variable^a^ (effect)	*df*	Reduction in deviance (scaled)	*p* ^b^
Courtship	Age	1	80.696	<2.2 × 10^−16^
	Condition	2	58.484	1.997 × 10^−13^
	Age × condition	2	36.199	1.379 × 10^−08^
Copulation	Age	1	73.286	<2.2 × 10^−16^
	Condition	2	43.496	3.588 × 10^−10^
	Age × condition	2	22.144	1.554 × 10^−05^
Leg vibration	Age	1	99.231	<2.2 × 10^−16^
	Condition	2	10.637	0.004901
	Age × condition	2	0.75616	0.6852
Courtship duration	Age	1	15.458	8.431 × 10^−05^
	Condition	2	7.7898	0.02034
	Leg vibration	1	59.339	1.327 × 10^−14^
Copulation duration	Age	1	7.6750	0.005599
	Condition	2	13.842	0.0009869
	Age × condition	2	3.6340	0.1625

^a^Full models included age and condition as explanatory variables, except for courtship duration that included an additional variable, leg vibration. Effect of interaction term was examined by adding it to the models involving age and condition

^b^Significance of reduction in deviance, examined using likelihood ratio tests

Although the courtship rate was already at its maximum level with 5-day-old males, the complexity of their courtship behavior was different from that of 7-day-old males. There was a significant difference in leg vibration rate between 5- and 7-day-old males (Fig. 4, condition 1; reduction in deviance = 4.66, *df* = 1, *p* = 0.031), showing that expression of complex courtship behavior required more time than maturation of mating activity itself. Courtship duration was dependent on occurrence of leg vibration. In cases where leg vibration was not performed, courtship duration was initially short, then became longer as males got older (Fig. 5a, condition 1). Even at age of 7 days, however, courtship duration was less than 200 s. In contrast, courtship duration was as long as 200 s at any age when leg vibration was performed (Fig. 5b). Combined with our previous observation showing that leg vibration was performed at the end of a courtship bout (Setoguchi et al. 2014), this result suggests that leg vibration was performed only at the end of a courtship bout with a certain duration (200 s in this case).

These results clearly demonstrate that the courtship behavior of *D. prolongata* males changed during early adult phase. In particular, expression of complex courtship behavior required more time than maturation of mating activity itself. Courtship of younger males was immature and simple; they attempted to copulate with shorter courtship duration and less leg vibration. In addition to courtship behavior, copulation duration also showed an age-dependent change by decreasing as males got older (Fig. 6, condition 1).

### Effect of social condition

Understanding the developmental changes in courtship behavior, we then examined the effect of social condition at each age by putting two males in a single staging vial. Social experience reduced mating activity, with courtship rate decreased at all ages (Fig. 2, condition 2), and copulation rate decreased accordingly (Fig. 3, condition 2). A similar result was observed for leg vibration rate; although the effect of the experimental condition was statistically significant only in 7-day-old males, social experience reduced leg vibration rate (Fig. 4, condition 2). Courtship duration was not different between conditions 1 and 2 except for in 7-day-old males that performed leg vibration, whose courtship duration was longer when they were staged in social condition (Fig. 5b, condition 2). This result may suggest that males hesitated to perform leg vibration, anticipating interference by rival males (see “Discussion”). Copulation duration did not decrease with age in social condition (Fig. 6, condition 2). Consequently, the copulation duration of socially experienced males was significantly longer in 7-day-old males.

### Plasticity of social effect

**Fig. 1:**
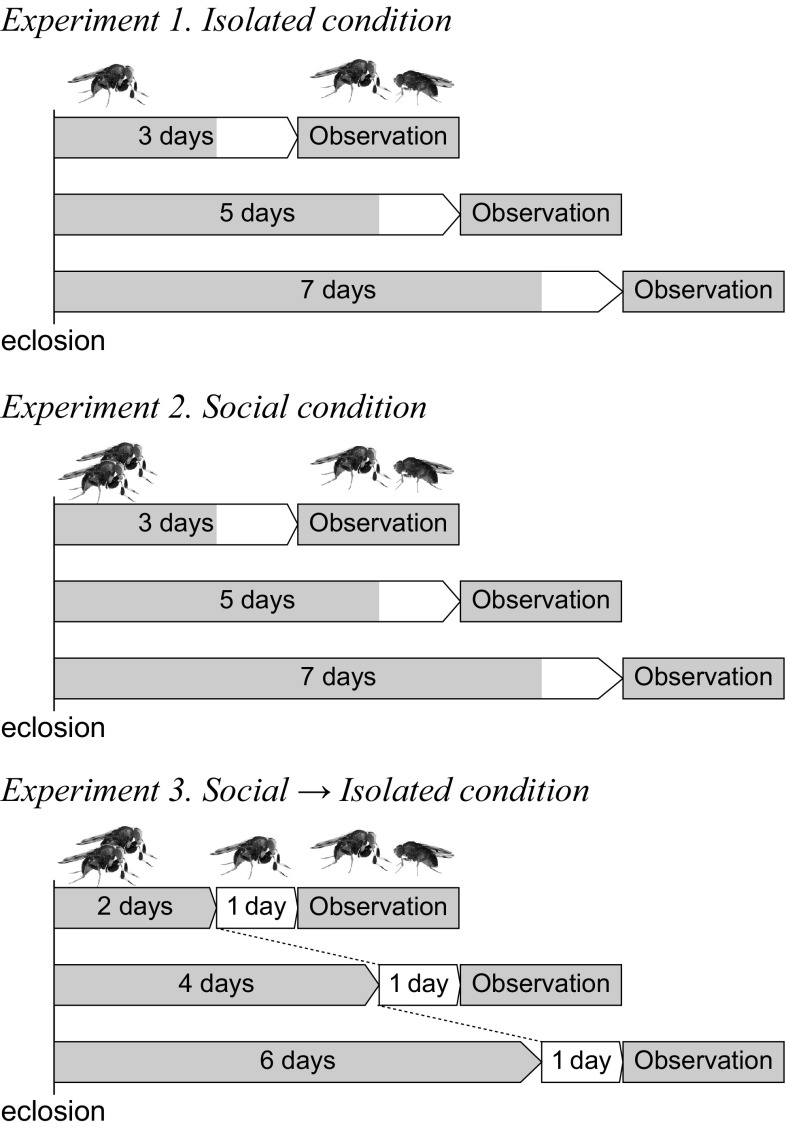
Experimental design. Newly emerged males were staged in three different conditions for 3, 5, or 7 days before individual observation of mating behavior with a 7-day-old female. Shading indicates food condition; *gray* with food, *white* without food (starvation treatment)

As seen above, males that experienced social condition behaved as if they were younger males staged in isolated condition. An alternative explanation is possible, however; i.e., it could be due to plastic behavioral adjustment of males that anticipated interference by rival males or an increased risk of postcopulatory competition (see “Discussion”). To examine whether the social effect on courtship behavior was reversible, we applied a combination of the two conditions by shifting males from social to isolated condition (Fig. 1, condition 3).

**Fig. 2:**
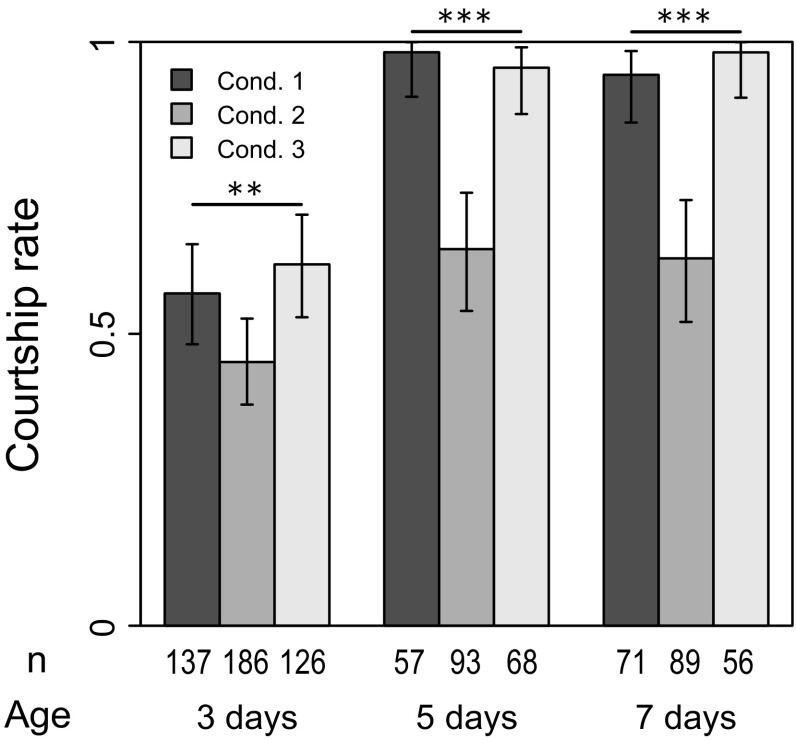
Proportion of males showing any courtship during the observation period (courtship rate). *Condition 1* isolated (single male in a vial), *condition 2* social (two males in a vial), *condition 3* social → isolated. *Error bars* represent 95 % confidence interval by binomial test. **p < 0.01, ***p < 0.001 (LRT using GLM)

**Fig. 3:**
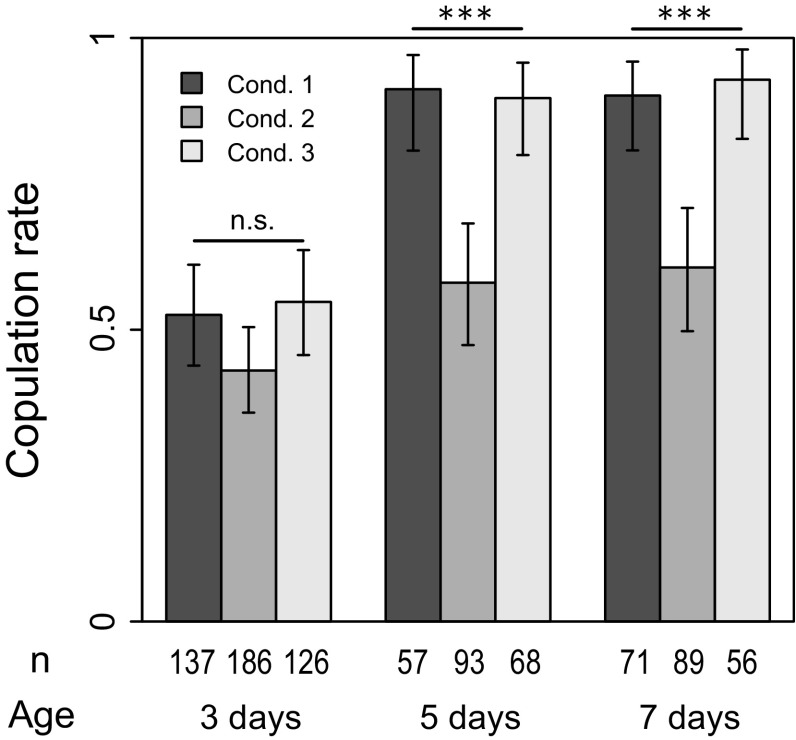
Proportion of pairs that copulated during the observation period (copulation rate). *Condition 1* isolated (single male in a vial), *condition 2* social (two males in a vial), *condition 3* social → isolated. *Error bars* represent 95 % confidence interval by binomial test. *n.s. p* > 0.05, *p < 0.05, **p < 0.01, ***p < 0.001 (LRT using GLM)

**Fig. 4:**
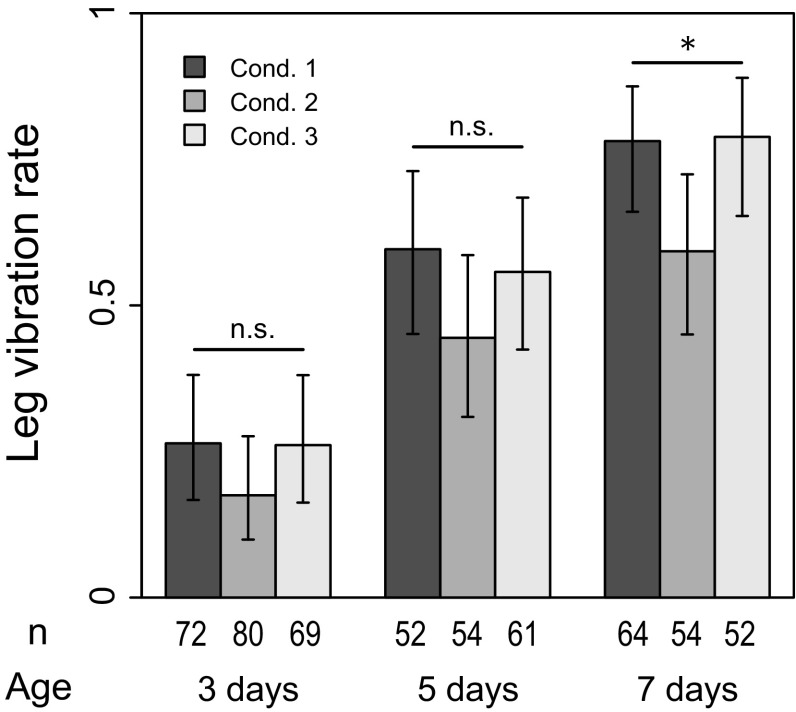
Proportion of pairs that performed leg vibration among pairs that copulated (leg vibration rate). *Condition 1* isolated (single male in a vial), *condition 2* social (two males in a vial), *condition 3* social → isolated. *Error bars* represent 95 % confidence interval by binomial test. *n.s. p* > 0.05, *p < 0.05, **p < 0.01, ***p < 0.001 (LRT using GLM)

**Fig. 5:**
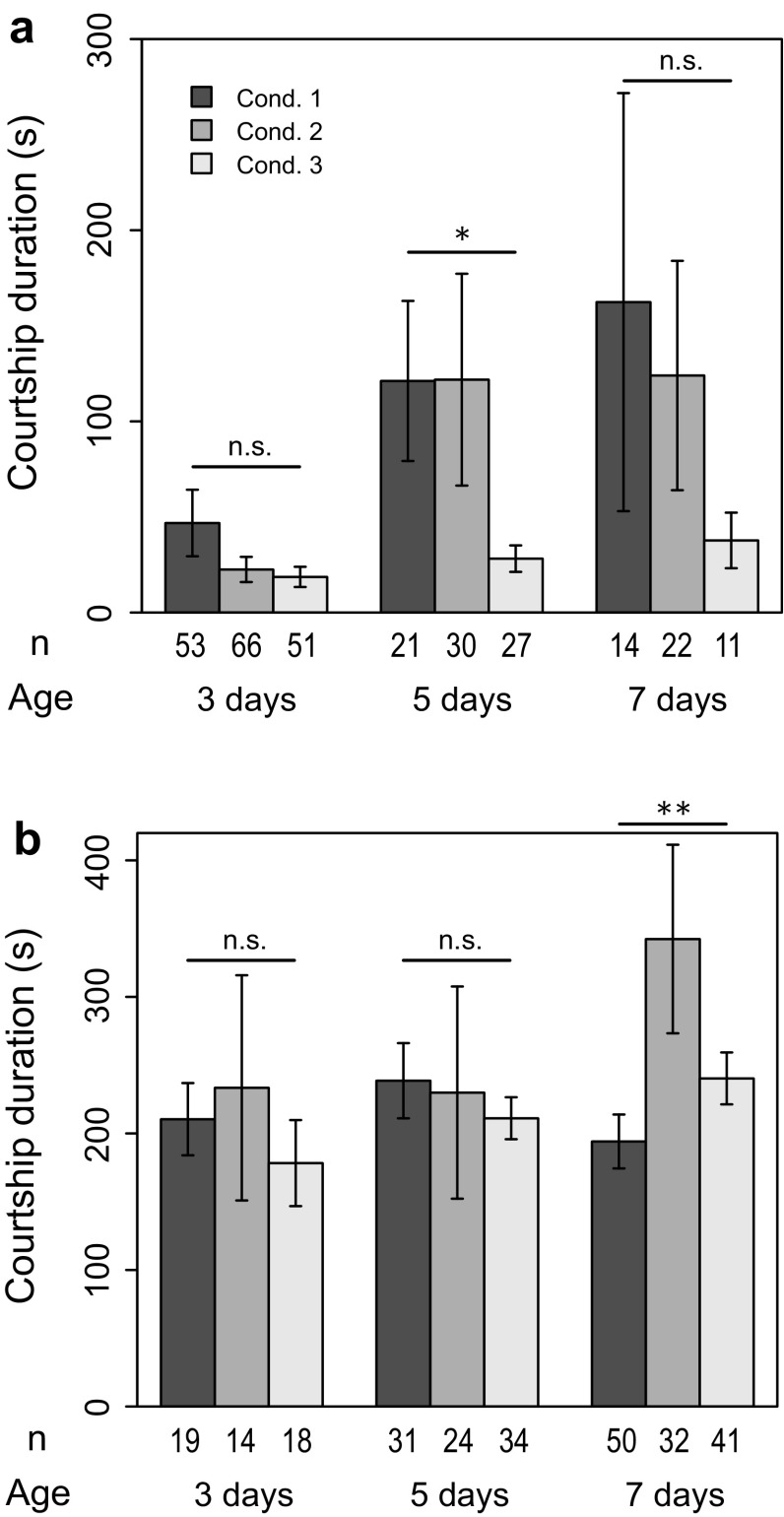
Duration of courtship among pairs that copulated. *Condition 1* isolated (single male in a vial), *condition 2* social (two males in a vial), *condition 3* social → isolated. *Bars* represent means, and *error bars* represent standard error. **a** Duration of courtship in pairs that copulated without leg vibration. **b** Duration of courtship in pairs that copulated with leg vibration. *n.s. p* > 0.05, *p < 0.05, **p < 0.01, ***p < 0.001 (LRT using GLM)

**Fig. 6:**
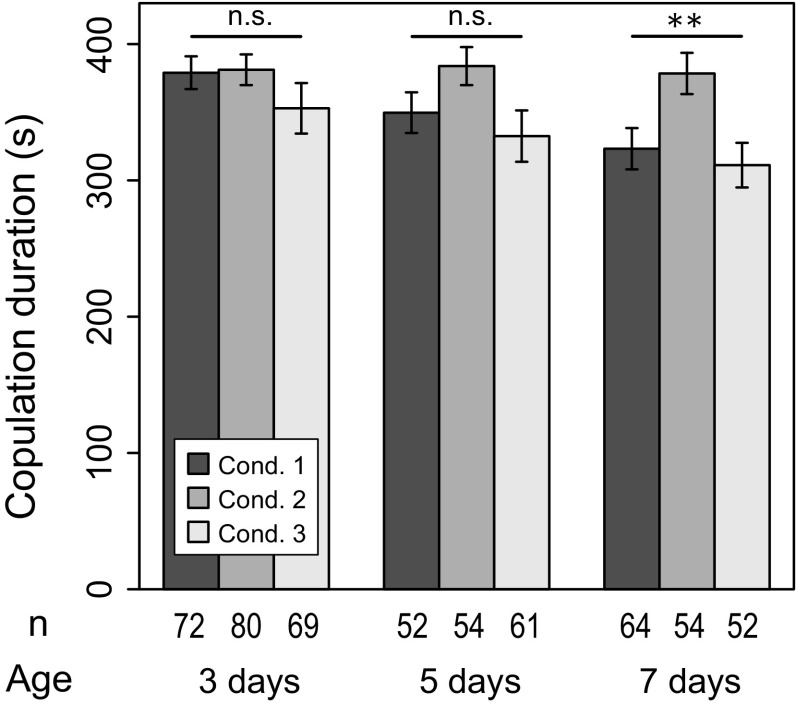
Duration of copulation. *Condition 1* isolated (single male in a vial), *condition 2* social (two males in a vial), *condition 3* social → isolated. *Bars* represent means, and *error bars* represent standard error

Social effect was completely erased by 1 day of isolation, and most of the results from condition 3 were comparable to those of condition 1 (Figs. 2, 3, 4, 5, 6). Therefore, the social effect was reversible and probably did not suppress behavioral development. This could indicate a plastic response of males to reduce investment in courtship (see “Discussion”). It was also suggested that the social effect did not accelerate behavioral development because all results from condition 3 did not exceed those of condition 1. However, it should be noted that courtship duration without leg vibration was an exception, being significantly shorter in males under condition 3 (Fig. 5a). The reason is currently unknown.

## Discussion

### Development of courtship behavior during early adult phase

Developmental changes in mating behavior in *Drosophila* have been studied as a part of the sexual maturation process; therefore, mating activity has attracted more attention than the structure of courtship behavior itself (Boake and Adkins 1994; Ford et al. 1988; Long et al. 1980; Moulin et al. 2001; Pitnick et al. 1995; Ruhmann et al. 2016; Wijesekera et al. 2016). In this study, not only mating activity but also the complexity of courtship behavior was shown to exhibit developmental changes during early adult phase in *D. prolongata*. Courtship behavior changed in at least two aspects: courtship duration and involvement of leg vibration. As males became older, they spent more time on courtship and performed leg vibration more frequently. Such age-dependent changes resulted in the more complex courtship performed by mature males. Younger males tended to attempt copulation after shorter courtship duration and with less leg vibration. Their courtship was different from the complex courtship performed by mature males, and appeared “hasty” and “rude.” In this study, even with such immature courtship, young males could copulate with the SaPa010 females. However, SaPa010 females are known to accept copulation practically without any courtship, in contrast to the majority of *D. prolongata* females that are much more reluctant to accept copulation (Setoguchi et al. 2014; Kudo et al. 2015). Therefore, young males would be rejected by most females under natural conditions.

Thanks to the complex courtship behavior involving leg vibration, behavioral development is easily recognized in *D. prolongata*. However, developmental changes were also significant in courtship duration, which may be comparable to “copulation latency” or “mating latency,” a parameter routinely used in studies of other *Drosophila* species (measured as the time from initiation of courtship to copulation). Although copulation latency is usually considered as a parameter representing male attractiveness to females, our results showed that courtship duration (≈copulation latency) was also able to reflect the intrinsically generated behavioral pattern of males by using high-receptivity females such as SaPa010, suggesting that it might be possible to find age-dependent changes of courtship behavior even in other *Drosophila* species with more stereotyped courtship such as *D. melanogaster*.

### Social experience influenced the structure of courtship behavior

In our previous study, males of *D. prolongata* were shown to change their courtship behavior in the presence of rival males by reducing the use of leg vibration, which is vulnerable to eavesdropping by rivals that would intercept the courted females immediately after leg vibration was performed (Setoguchi et al. 2015). In the present study, males that had been maintained with other males performed less leg vibration even when there were no other males in the mating arena. They also spent more time on courtship before performing leg vibration, implying that they hesitated in anticipation of interference by rivals. These results clearly demonstrate that social experience, not current social condition (presence of rivals), influenced courtship behavior. Similar effects have been reported in other *Drosophila* species; For example, the copulation rate was reduced by staging in social condition in *D. subobscura* (Lizé et al. 2014). Copulation duration was also shown to increase by exposure to rivals in *D. melanogaster* (Bretman et al. 2009) and four other species (Lizé et al. 2012). However, these examples do not involve remodeling of courtship behavior. Our results present a unique example, in which the structure of courtship behavior itself was changed by social experience.

It is noteworthy that, although copulation duration increased with social experience in accordance with the other studies mentioned above, the present study of *D. prolongata* also revealed that younger males copulated for longer, and copulation duration decreased along aging in isolated condition. In other words, socially conditioned males did not extend their copulation duration, but they kept it as long as that of younger males. This result was the opposite to a proposed hypothesis, in which aging has been suggested as a cause of elongation of copulation duration in *D. subobscura* (Lizé et al. 2014).

### Reversible effect of social condition

As discussed above, the effect of social experience appeared to work in the direction to suppress expression of courtship behavior. This effect was reversible; only 1 day of isolation was enough to fully recover the behavioral structure as observed in males maintained in isolated condition throughout the staging period. This result suggests that the social effect represents behavioral plasticity, by which males adjust their investment in courtship when increased risk of male–male competition is expected (e.g., female interception and sperm competition). Learning and memory or other physiological mechanisms are likely to underlie this behavioral plasticity. It should be noted that development of complex courtship behavior was not promoted at least by male–male interaction because behavioral recovery was exactly to the same level as observed in condition 1. The exception was courtship duration without leg vibration, which became longer as males became older in conditions 1 and 2, but stayed short in condition 3, suggesting an irreversible effect of social condition on courtship behavior. No reasonable explanation is currently available for this difference.

## Conclusions

Age-dependent changes in courtship behavior of *D. prolongata* were examined. Expression of complex courtship required a longer developmental period than the simpler form. Furthermore, social experience with rival males plastically suppressed expression of complex courtship. These results provide insight into the mechanisms underlying the interaction between social experience and the developmental process of behavior, and serve as a foundation for further studies.
